# Fracture Analysis of Short-Scale Corroded/Healthy Reinforced Concrete Beams under Bending Using Acoustic Emission

**DOI:** 10.3390/ma16217011

**Published:** 2023-11-02

**Authors:** Mouhamadou Mountakhah Seye, Yuma Kawasaki, Ejazulhaq Rahimi

**Affiliations:** 1 Graduate School of Science and Engineering, Ritsumeikan University, Kusatsu 525-0058, Japan; gr0448vi@ed.ritsumei.ac.jp; 2 Department of Civil and Environmental Engineering, Ritsumeikan University, Kusatsu 525-0058, Japan; yuma-k@fc.ritsumei.ac.jp

**Keywords:** acoustic emission, bending test, electrical corrosion, reinforced concrete beam, location analysis

## Abstract

In this work, the acoustic emission (AE) technique is used to evaluate the fracture process of corroded and healthy reinforced concrete (RC) beams subjected to a monotonic bending test. In fact, many researchers have conducted laboratory experiments considering different conditions to perform rebar corrosion monitoring in RC structures using the AE method. However, previous studies have not investigated the evolution of the bending performance of RC beams at different corrosion degrees, considering the interaction of single rebar with concrete. In this study, healthy and electrically corroded RC beams are evaluated, considering different corrosion levels. The analysis of the moving average of the AE maximum amplitudes was consistent to distinguish four stages of mechanical behavior that the healthy, and corroded specimens with low and medium corrosion levels of 0.9% and 3.2% experienced up to failure. Three damage stages were identified in the case of a high corrosion level of 9.3%. Then, the AE maximum amplitudes were suitable to establish an efficient clustering, which enabled the classification of the fractures into minor, medium, and major classes. Furthermore, the digital analysis method proposed in this study was suitable to visually reveal the influence of the preexisting corrosion-induced damages on the bending failure process of the RC beams.

## 1. Introduction

Numerous studies focusing on rebar corrosion monitoring in RC structures using acoustic emission (AE) techniques have been conducted. Some of these studies involved the evaluation of the mechanical performances of corroded RC structures using AE techniques. However, utilizing the AE method, it is also important to examine the correlation between the rebar corrosion process and the mechanical performances of corroded RC structures.

AE techniques are considered one of the non-destructive testing (NDT) methods. It has demonstrated its usefulness in assessing the damage to RC caused by the corrosion process and in determining the level of corrosion-induced damage. Chloride attack is one of the major causes of rebar corrosion in reinforced concrete. In salty environments, the rate of deterioration increases rapidly when chloride-induced corrosion occurs in reinforcing bars, which can have a serious impact on their overall performance. In many studies, AE parameters such as AE hits and energy are used to characterize the corrosion process in steel reinforcement members [1,2,3,4,5,6,7]. The AE sources are classified according to average frequency (AF) and RA value, which are both used to distinguish the failure types. Furthermore, the AE technique enables the location of the steel corrosion damage to be identified, as well as facilitating the detection of concrete cracking [8,9,10].

Hence, this research aimed to evaluate the failure mechanisms of corroded and healthy RC beams under bending loading using the AE technique in the context of the rebar corrosion process. Among several NDT methods, the choice to perform this work focused on the AE technique thanks to its advantages. In fact, the AE technique is a sensitive technique that can detect and locate active defects within a material, such as concrete. The AE sources are the deformation processes, such as cracks that grow, which release localized energy. Then, that released energy generates AE waves that are detected by sensors placed on the concrete surface. For this purpose, twenty specimens made with normal deformed rebar were arranged into five sets of four specimens each. Four sets were corroded to different degrees of corrosion, and another set was reserved as healthy specimens. The process of corrosion was electrically accelerated by applying a 200 mA current through the specimens’ rebar, which was partially immersed in a 3% chloride concentration saltwater along the rebar location. Thereafter, bending loading tests were conducted on all specimens. Both the corrosion process and bending tests were conducted using an AE monitoring technique, and the results were analyzed to determine the bending behaviors of RC beams during their failure process. Based on the experimental results, it was confirmed that the corrosion of rebar substantially affected the performance of the RC beams and had a considerable impact on their failure process. Moreover, corrosion-induced cracks often lead to the initiation of concrete failure during the bending loading.

## 2. Literature Review

Various NDT techniques have been used in concrete structures. He et al. used a modified non-contact electrical resistivity measurement to evaluate the formation factor of mature cement-based materials [11]. They also employed the electrical resistivity and GPR measurement methods to evaluate the curing effectiveness of field concrete slabs [12]. Zhou et al. used digital image correlation (DIC) and wavelet packet analysis techniques to evaluate the initiation, development, and evaluation of micro-damages occurring in concrete beams [13]. Among various NDT techniques, the AE method is considered the only one that enables passive and global monitoring of active defects in diverse materials and structures without affecting their workability and can even be combined with other NDT methods. Jing et al. [14] combined the AE technique, digital speckle correlation method, and numerical simulations to investigate the micro-mesoscopic creep damage and the failure mechanism of sandy mudstone. Xu et al. [15] used the AE technique to elaborate a damage constitutive model describing the mechanical response of gypsum rock in humid conditions.

Many researchers have especially performed laboratory experiments considering different perspectives to study the characteristics of AE from RC members affected by rebar corrosion. For instance, Chen et al. used the AE technique to identify cracks in corroded RC columns subjected to eccentric loading [16]. Seye et al. used the AE technique to study the fracture performance of corroded RC cylinders subjected to compressive loading [17].

Nevertheless, few studies have investigated the correlations between corroded and healthy RC beams, applying AE techniques. Zheng et al. [18] studied the fractal characteristics and evaluated the damage process of corroded beams subjected to four-point bending tests using AE techniques. The analysis of AE ringing counts showed that the damage of concrete and the recorded AE signals could be closely correlated. Moreover, three damage stages could be observed: the first stage related to initiation of damage, the second stage corresponding to the damage evolution, and the third stage showing the growth of the continuous damage. Kawasaki et al. [19] performed an evaluation of RC beams damaged due to rebar corrosion using the acoustic emission technique. The results of the analysis of the AE parameter revealed a difference in the fracture process between the environments with low and high chloride concentrations. In specimens with a low chloride concentration, there was a high number of tensile cracks. However, in specimens with a high chloride concentration, there was a high number of shear cracks. Prem et al. [20] could distinguish four stages of mechanical behavior during the failure process of a long RC beam subjected to bending loading. The first stage corresponded to the microcracking, and the propagation of the localized cracks represented the second stage. The distributed flexural cracks followed by the occurrence of damage were, respectively, considered the third and fourth stages. The effects of longitudinal rebar on the AE parameters were similar. With the increase in the reinforcement ratio, an increase in the average and cumulative values of the AE parameters was noted. Zaki et al. [21] showed via experimental results that the loss of the ultimate strength of corroded reinforced concrete beams was related to the reduction in accumulated AE hits. According to their conclusion, the trend of cumulated AE hits could be attributed to the stress and cracks related to tension rebar, which had already been dissipated by corrosion. Abouhussien et al. [22] evaluated the bond behavior of corroded rebar in RC prism specimens subjected to pull-out testing using the AE method. The results revealed that there was a very close correlation between the cumulative number of AE hits and the AE signal strength related to the degradation of the bond between rebar and concrete caused by corrosion. Garhwal et al. [23] utilized the AE method to assess the behavior and the performance of large RC beams affected by corrosion under bending loading.

However, previous studies have not investigated the evolution of bending performance of RC beams at different corrosion degrees, considering the interaction of single deformed rebar with concrete. Therefore, using the AE technique, this study aims to assess the damage evolution in corroded and non-corroded small-scale RC beams fitted with single rebar. Four-point bending tests were performed on RC beams with different corrosion levels, and AE data were collected. These corrosion levels were, respectively, 0% as a healthy specimen, 0.9%, 3.2%, and 9.3%. These corrosion levels were designed to imitate, respectively, low, medium, and high rebar corrosion degrees in RC structures. First, the variation characteristics of the cumulative AE hits with loading were investigated and compared with the specimen condition regarding the corrosion level. Next, a phase of the damage evolution was established based on the maximum amplitude of recorded AE hits and the AE sources’ location inside the specimens. Subsequently, due to the use of small specimens fitted with single tension rebar, this work was able to accurately monitor the interaction between rebar and concrete fracture damage in RC beams under both healthy and corroded conditions, based on AE monitoring.

## 3. Materials and Methods

### 3.1. Specimens Confection

**Figure 1:**
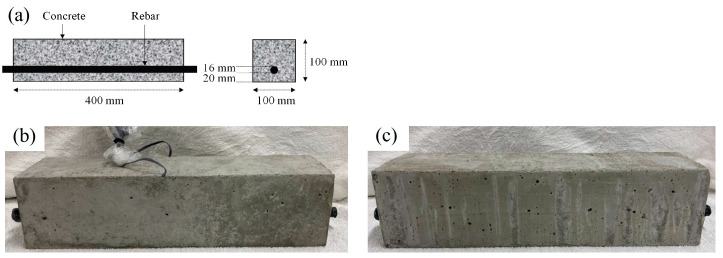
RC beam specimens. (**a**) Geometrical details; (**b**) specimen with rebar strain gauges; (**c**) specimen without rebar strain gauges.

In this study, 16 small-scale reinforced concrete beams with identical dimensions (100 × 100 × 400 mm^3^) were used. They were all fitted with a single deformed tension rebar with a diameter of 16 mm, located 20 mm from the bottom surface, as shown in Figure 1a. One specimen from each set included two strain gauges fixed at mid-span on the tension rebar (Figure 1b,c). To examine the mechanical properties of the concrete at 28 days and on the bending test day, six standard cylinders of plain concrete 100 mm in diameter and 200 mm high were made when constructing the beams. All specimens were prepared and cured under the same conditions. The mix proportions of the concrete are presented in Table 1. After casting the concrete, the RC specimens were moisture-cured under a shower using tap water for 28 days. Following this curing period, they were dried for seven days at room temperature and were then immersed in salt water with a 3% chloride concentration for seven days. This process promoted the penetration of chloride ions inside the specimens. Prior to the immersion in salt water, both ends of the RC beams were coated with epoxy resin to exclusively allow chloride ingress via the lateral sides in order to simulate salt attack in a real-world setting.

### 3.2. Corrosion Assessment Using Impressed Current Technique

**Figure 2:**
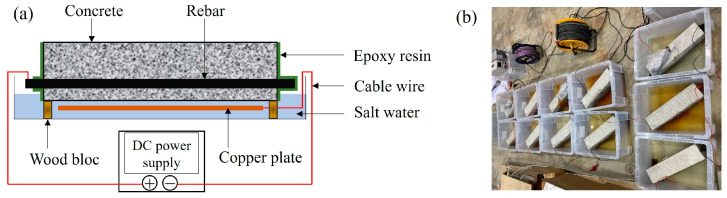
(**a**) Corrosion process principle. (**b**) Electrical corrosion process on RC specimens.

The sixteen RC beams were arranged into four sets of four specimens each. Three sets were corroded to different degrees of corrosion, and the other set was kept as non-corroded specimens. To obtain reasonable results within a realistic time frame, laboratory corrosion tests were conducted using an electrically accelerated process, often referred to as the impressed current technique. A 3% NaCl solution was used as an electrolyte and was representative of marine environments. The rebar was used as the anode, while a copper plate acting as the cathode was put along each axial rebar. The corrosion process was electrically accelerated by applying 0.2 A current, which was provided by a direct current power supply (Figure 2a,b). Each specimen was placed separately in a plastic container on two pieces of wood because wood is not affected by the redox reaction. The first set was corroded for 48 h, the second one for 96 h, the third one for 144 h, and the fourth set for 336 h. 

After performing electrically induced corrosion, rebar was chipped out from one specimen from the corroded sets to evaluate the corrosion level. Next, the corrosion products were chemically removed from the rebar surface via immersion in a di-ammonium hydrogen of 10% concentration, at which the temperature was kept constant at 60 °C for 24 h. Then, the rebar corrosion rate in each case was evaluated based on the JCI code (JCI-SC1, Corrosion evaluation method of steel in concrete). The maximum width of the corrosion-induced cracks was also measured on the corroded specimens. The first set was corroded with a corrosion level of 0.9%, the second one with 3.2%, and the third one with 9.3%. The average crack widths at the bottom surface of specimens were, respectively, 0.05 mm, 0.10 mm, and 0.50 mm, and were located next to the rebar.

### 3.3. Bending Test Using AE Technique

#### 3.3.1. AE Technique Approach

**Figure 3:**
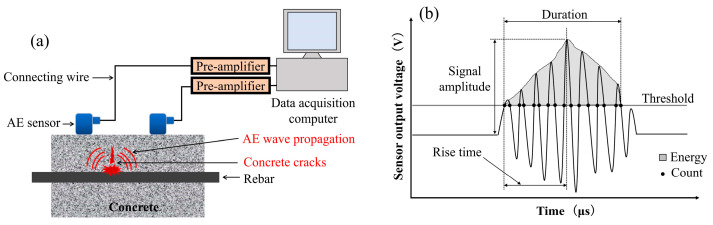
(**a**) AE acquisition principle. (**b**) Schematic showing parameters of an AE waveform.

The AE technique has been widely employed for monitoring both structural health and rebar corrosion, particularly regarding the evaluation of corroded RC structures [6,7,24,25,26]. This method was implemented to analyze the process and the progression related to concrete structures [27,28,29]. Steen et al. [30] proceeded to the localization and the characterization of corrosion-induced damage in reinforced concrete. The AE method is also applicable, feasible, and sensitive for the analysis and assessment of rebar corrosion in RC members. Moreover, there are two types of acoustic emission measurements: parameter analysis and signal-based analysis [29,31]. The principle of the AE method used in this study is shown in Figure 3a, and AE parameters are depicted in Figure 3b. After performing electrically induced corrosion on three sets of specimens, the corroded and non-corroded small RC beams were subjected to four-point bending load testing using the acoustic emission technique. In this study, the maximum AE amplitudes fluctuated from 40 dB to 100 dB.

#### 3.3.2. Bending Test Process

**Figure 4:**
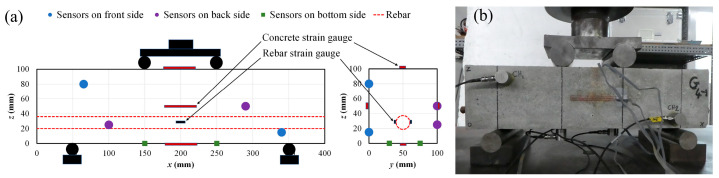
(**a**) Schematic of bending load testing on RC beam specimens, (**b**) bending load testing on RC beam specimen using AE technique.

After performing electrically induced corrosion, the corroded and non-corroded specimens were subjected to four-point bending load testing up to failure. The flexural load test was carried out on three specimens from each set using a universal testing machine. Each specimen was supported at both ends. Furthermore, AE data and rebar and concrete strain data were acquired and recorded using one specimen prepared from each set. Six AE sensors of 150 kHz of resonance were mounted on the data acquisition specimens via the use of wax as a coupling agent. They were mounted two-by-two on three lateral surfaces of the specimens to enable a three-dimensional AE source location. Figure 4a provides the details on the distances between the supports and the loading points, and the coordinates of the AE sensors are listed in Table 2. Each sensor was connected to a preamplifier, which was connected to the acquisition computer. The AE sensors, the preamplifiers and the acquisition system are manufactured by MISTRAS Group, Princeton Junction, NJ, USA. The type of the sensors are (R15α) and the category of the preamplifiers is 2/4/6—Switch Selectable Gain Single-Ended and Differential. These voltage preamplifiers are fitted with switch-selectable gain ranges of 20, 40, and 60 decibels. As for the acquisition system, the AEwin version 2014 was used. Concerning event counting, the dead time was set to 2 ms, and the threshold level was set to 40 dB gain. The loading was single and monotonic, and the speed was set to 0.2 kN/s. The bending process is presented in Figure 4b.

#### 3.3.3. AE Analysis Method

After performing AE measurement during bending testing, all AE data with an energy equal to 0 were deleted. The AE energy is defined as the area of an AE signal above the threshold and represents its size. Therefore, a minimum non-null energy failure was considered. Additionally, all AE data with a count equal to 1 were deleted as well. As the AE count is related to the frequency at which the AE signal amplitude exceeds a predetermined evaluation threshold within the AE signal, the signals that did not exceed the threshold were not identified as AE signals. Next, the remaining data were explored to proceed with the AE analysis, using the methods present below.

**Figure 5:**
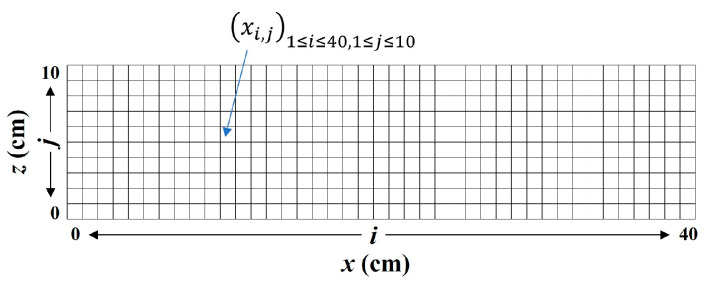
Cells gridlines system for AE sources concentration analysis.

- Fracture scale classification: Fractures were classified in terms of the intervals related to the maximum amplitudes of AEs generated inside the specimens during the bending test. The mean (µ) and standard deviation (*σ*) of the maximum amplitudes were used. Therefore, three intervals, *I_minor_*, *I_medium_*, and *I_major_*, were established, respectively, related to minor fracture, medium fracture, and major fracture. They are presented in Equations (1)–(3), and the results are shown in Table 3.
  (1) $$I_{minor}=[40,µ-\sigma -1]$$
  (2) $$I_{medium}=[µ-\sigma ,µ+\sigma ]$$
  (3) $$I_{major}=[µ+\sigma +1,100]$$
- RA values and average frequencies parameters: Based on these three fracture scale intervals, a classification of the RA values and the average frequencies was performed as well to classify the cracks mode in shear-type and tensile-type. The RA values and the average frequencies were obtained from the AE parameters [32,33] using Equations (4) and (5) as follows:
  (4) RA=Rise timeMaximum amplitude
  (5) AF=CountDuration
  where Rise Time is the time from an AE signal’s first threshold crossing to its peak; Maximum amplitude is the largest voltage peak value in an AE signal waveform; Count is the number of times an AE signal crosses the detection threshold; and Duration is the time that elapses from the crossing of the first threshold to the end of the last threshold crossing of an AE signal from the AE threshold.
- Failure stages classification and AE sources’ location: The moving average of the maximum AE amplitudes, generated during bending testing, was explored to identify the different failure stages with time history. Therefore, a range of 50 was used to set the moving average of the maximum amplitude of AE events. Furthermore, to evaluate the concentration of AE sources generated during the bending process, a three-dimensional representation was adopted. Therefore, square cells with the dimensions of 10 mm × 10 mm were designed on the specimens’ sides surface via the x, y, and z axis. In each case, the maximum value $Max\left(x_{i,j}\right)$ of the concentration $x_{i,j}$ of AE sources in a cell via the front side of the specimen were determined, where i and j are, respectively, the position of a cell in the x axis from 1 to 40, and in the z axis from 1 to 10 (Figure 5). Then, the minimum of those four maxima $Min\left[Max\left(x_{i,j}\right)\right]$, presented in Table 4, were considered to define four categories of AE sources concentration in a three-dimensional representation (Equations (6)–(9)).
  (6) n=0
  (7) $$0<n\leq Min[{Max\left(x_{i,j}\right)}_{1\leq i\leq 40,1\leq j\leq 10}]$$
  (8) $$50\%Min[{Max\left(x_{i,j}\right)}_{1\leq i\leq 40,1\leq j\leq 10}]<n\leq Min[{Max\left(x_{i,j}\right)}_{1\leq i\leq 40,1\leq j\leq 10}]$$
  (9) $$n>Min[{Max\left(x_{i,j}\right)}_{1\leq i\leq 40,1\leq j\leq 10}]$$
  where *n* is the concentration of AE sources in a cell in a three-dimensional representation.

## 4. Results and Discussions

### 4.1. Compressive and Tensile Bending Stresses and Rebar Strain

**Figure 6:**
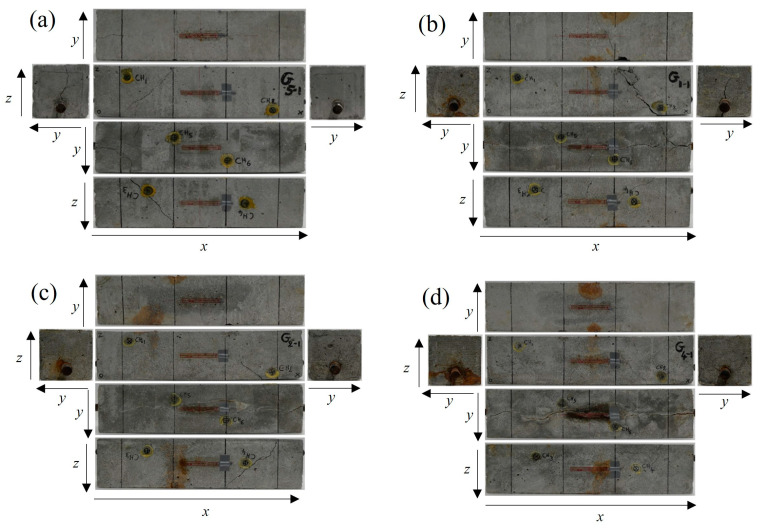
Failure profiles of the specimens at the end of bending tests. (**a**) Healthy condition; (**b**) 0.9% corrosion level; (**c**) 3.2% corrosion level; (**d**) 9.3% corrosion level.

**Figure 7:**
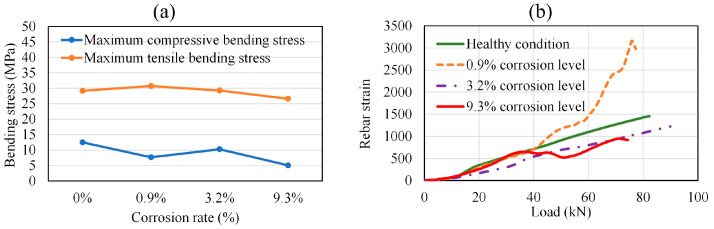
(**a**) Compressive and tensile bending stresses. (**b**) Rebar strain with load.

In Figure 6a–d, the failure profiles of the RC beams at the end of bending tests are presented for the healthy condition and the corroded cases. As all the specimens have a rectangular parallelepiped shape, the six faces of each specimen are presented for the visual observation of the damages resulting from the bending process. Therefore, the bending damage on different sides of the specimens can be observed. The extent of cracks observed in Figure 6a–d are further used in the representation of the crack’s location in Section 4.5, in correlation with preexisting corrosion-induced cracks. Figure 7a reveals that the compressive stress and tensile stress performance during bending testing tended to decrease with an increase in the degree of rebar corrosion inside the specimens. Many researchers have reported that bond strength between rebar and concrete in RC structures increases at a low level of corrosion. This phenomenon is attributable to an increase in rebar roughness and its confinement by concrete. However, a severe level of corrosion causes a sharp reduction in bond strength due to rebar slippage [34,35,36]. Therefore, the rebar strain tended to increase at the 0.9% corrosion level, regarded as a low degree of corrosion, then subsequently decreased as the corrosion level increased. At the 9.3% corrosion level, regarded as a severe degree of corrosion, the nonlinear irregular fluctuation of rebar strain was attributable to its intermittent slippage via the concrete. This was due to the formation of rust around the rebar, which caused the bond strength between the rebar and concrete to weaken (Figure 7b).

### 4.2. AE Hits and Load

**Figure 8:**
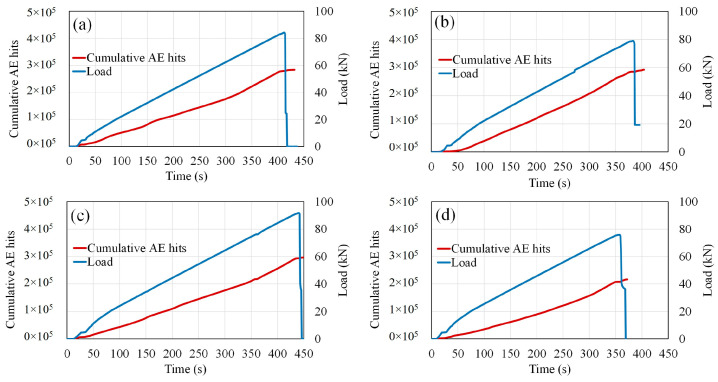
Cumulative AE hits and load during bending test: (**a**) healthy condition; (**b**) 0.9% corrosion level; (**c**) 3.2% corrosion level; (**d**) 9.3% corrosion level.

Globally, the cumulative AE hits increased gradually with the load in the healthy condition and in the corroded cases. Compared to the healthy condition, the interaction between the load rate and the generated cumulative AE hits did not show a significant difference for the 0.9% and 3.2% corrosion level cases, which can be considered low corrosion degrees. However, an increase in the corrosion level up to 9.3% is accompanied by a drop of the maximum load of 10%, a drastic drop in the number of generated AE hits of 24%, and a decrease in the time up to the failure of 13%. These findings were attributable to the formation of rebar corrosion inside the specimens, which resulted in differences in the AE signals generated during the bending process. Therefore, a severe increase in corrosion is accompanied by failure by a drop in the maximum load, a drop in the number of recorded AE hits, and a decrease in time to failure (Figure 8a–d). Zaki et al. [21] found a similar interaction between the accumulated AE hits and the corrosion level of RC beams under the bending process, with a loss of ultimate strength related to the reduction in accumulated AE hits. This trend of cumulative AE hits was attributed to the stressing and cracking at the tension rebar, which was already dissipated by preexisting corrosion damages. Therefore, they considered this declining trend as an alert to severe preexisting damages. Similar findings were also provided by Garhwal et al. [23], indicating that a significant drop in cumulative AE hits was observed when the corrosion level increased in larger-scale RC beams subjected to flexural loading. 

### 4.3. Failure Stages Classification with Maximum AE Amplitude

**Figure 9:**
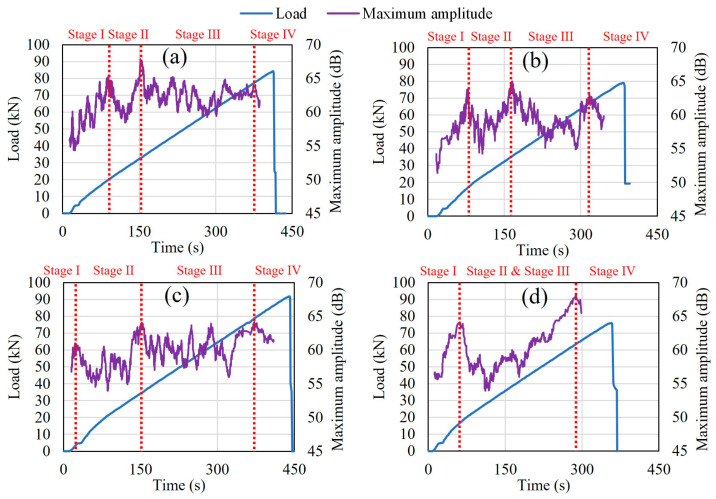
Failure stages classification with AE maximum amplitude. (**a**) Healthy condition; (**b**) 0.9% corrosion level; (**c**) 3.2% corrosion level; (**d**) 9.3% corrosion level.

Figure 9a–d presents the moving average of the maximum AE amplitude generated with load versus time during bending testing for each case (healthy condition, 0.9%, 3.2%, and 9.3% corrosion rate cases). A range of 50 was used to set the moving average of the maximum amplitude of AE events. The pattern of the development of the fracture process could be classified in terms of microcracks and macrocracks in the concrete structure during the bending process. Therefore, four stages of damage in the fracture process were identified in correlation with the fluctuation in the maximum amplitude of AE hits. From the beginning of loading, the fluctuation in the maximum amplitude showed a tendency to increase up to the first peak, defined as stage I, during which nucleation of cracks occurred. Then, there was a decreasing trend in maximum amplitudes followed by an increasing trend up to the second peak, defined as stage II. During the second stage, macrocracks began to form and grow from the bottom area of the specimens between the two lower supports toward the upper areas due to the deformation process. Moreover, while the load increased, no new cracks appeared in the specimens. Stage III, during which the macrocracks opened, corresponded to the fluctuation of the maximum amplitudes from the limit of stage II up to the last peak. Finally, stage IV corresponded to the last decreasing trend in the maximum amplitudes, at which point failure occurred. In addition, from the healthy condition up to the 3.2% corrosion level, there was still a time lapse between the formation of macrocracks and their opening period, which made stages II and III distinguishable. However, those stages merged at the 9.3% corrosion level because they occurred simultaneously (Figure 9d). These findings regarding severe corrosion conditions are supported by the results of Zheng et al. [18], even using bigger scale RC beams fitted with multiple bars and with three damage stages determined during bending tests. They also found that the damage to the uncorroded and lightly corroded beams occurred mainly in the final stage, while the damage to the severely corroded beam occurred earlier, prior to the final stage. 

### 4.4. RA Values and Average Frequencies Parameters

**Figure 10:**
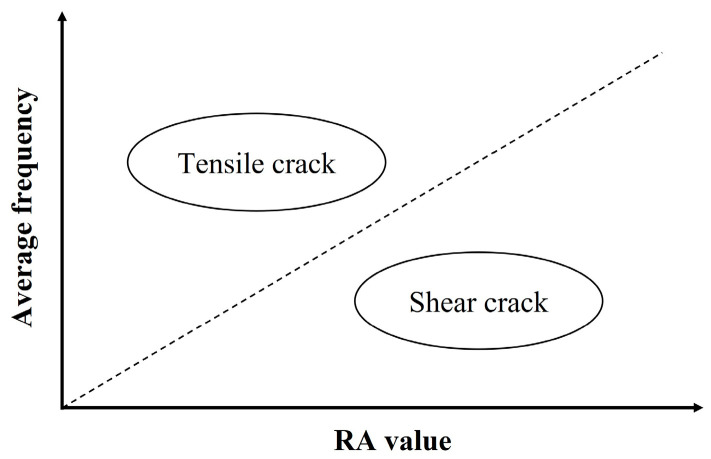
Conventional crack classification in JCMS-IIIB5706 code.

**Figure 11:**
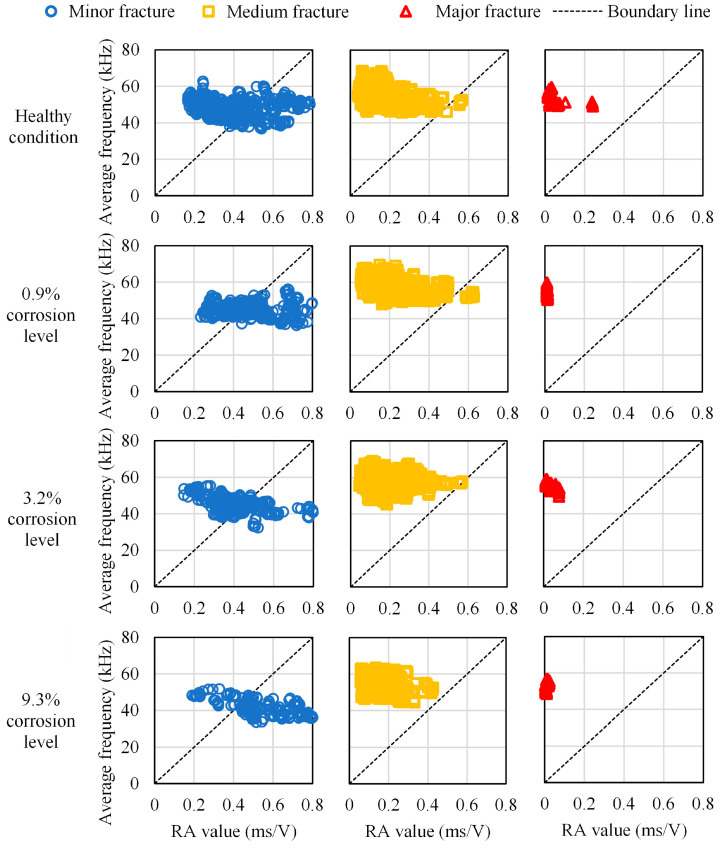
Relationship between RA value and average frequency in different cases with different fracture scales.

As illustrated in Figure 10, crack types can be classified based on the relationship between the RA value and the AF, according to the JCMS-IIIB5706 code. A tensile-type crack is referred to as an AE signal with high AF and low RA value. At the same time, a shear-type crack is designated by a low AF value and high RA value. These criteria are used to classify AE data recorded during the bending process. Based on these three fracture scale intervals defined in Section 3.3.3, a cross-analysis with the RA values and the average frequencies provided another classification, including damage scales and cracks mode, which are presented in tensile-type and shear-type. Although there is not yet a standard method for fixing the boundary line in the classification of RA values and average frequencies to identify tensile-type and shear-type damages, that classification made possible a comparative analysis of the fracture modes between the healthy condition and the different corroded cases. Therefore, the results from the relationship between RA-values and average frequencies show that the higher the corrosion level is, the more medium fractures migrate from predominantly the tensile type to the shear type, while the rate of medium fractures fluctuates inversely until being generated exclusively by the tensile type. Moreover, major fractures follow the same fluctuation as medium fractures Figure 11.

### 4.5. AE Sources and Crack Patterns

**Figure 12:**
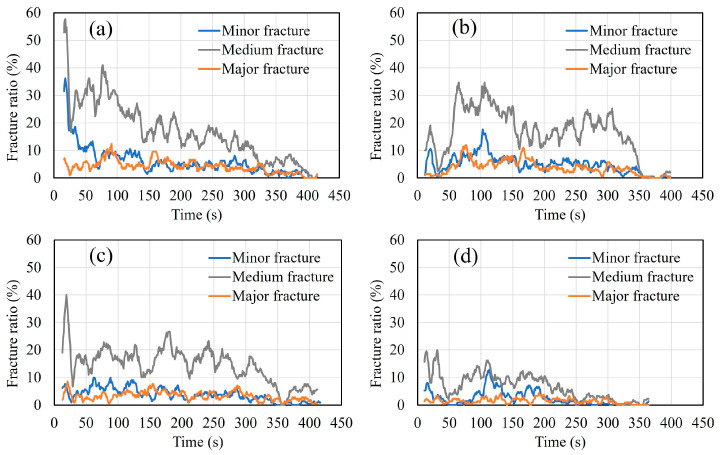
Progressive ratio of generated AE sources during the bending process with time history. (**a**) Healthy condition; (**b**) 0.9% corrosion level; (**c**) 3.2% corrosion level; (**d**) 9.3% corrosion level.

**Figure 13:**
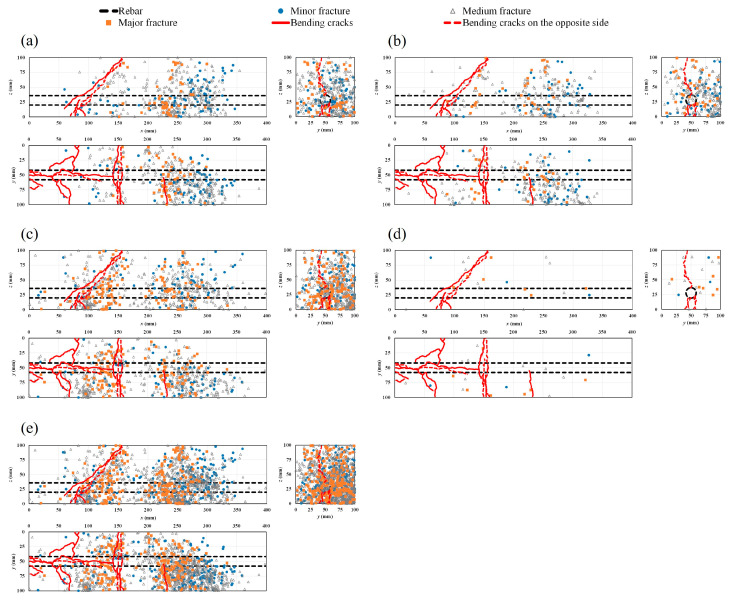
Location of AE sources and crack patterns in the healthy condition: (**a**) stage I; (**b**) stage II; (**c**) stage III; (**d**) stage IV; (**e**) all stages combined.

**Figure 14:**
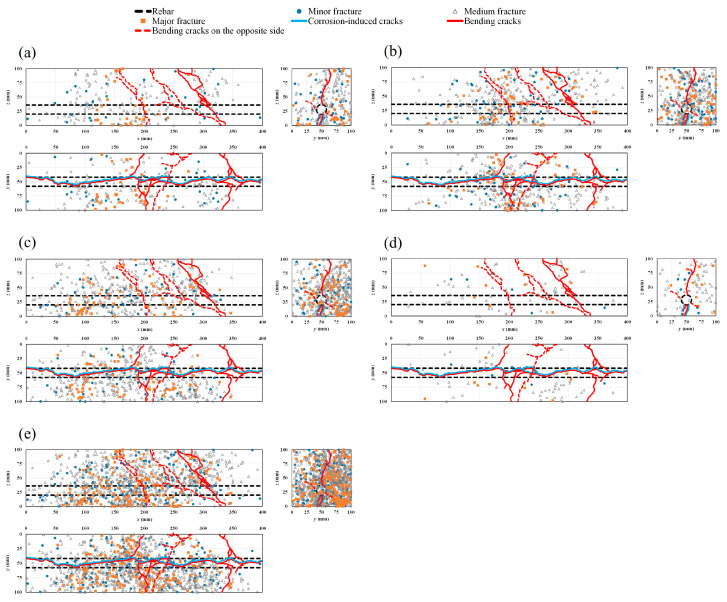
Location of AE sources and crack patterns for 0.9% corrosion level: (**a**) stage I; (**b**) stage II; (**c**) stage III; (**d**) stage IV; (**e**) all stages combined.

**Figure 15:**
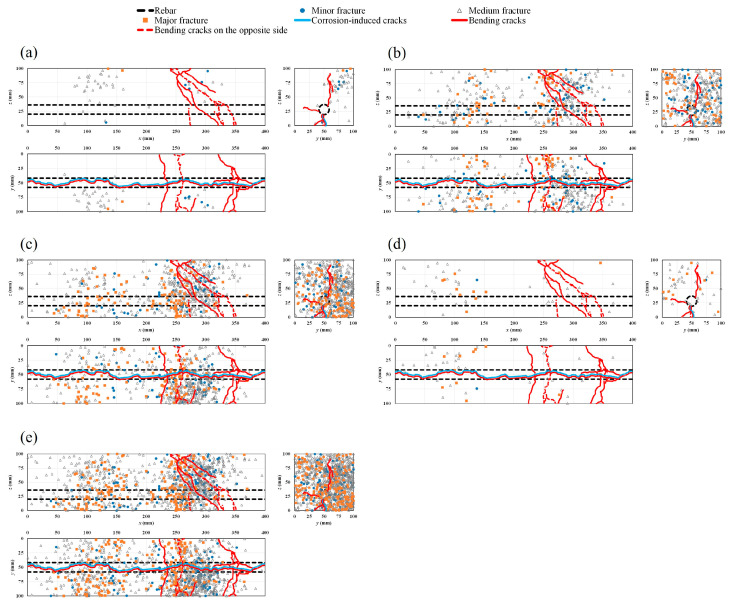
Location of AE sources and crack patterns for 3.2% corrosion level: (**a**) stage I; (**b**) stage II; (**c**) stage III; (**d**) stage IV; (**e**) all stages combined.

**Figure 16:**
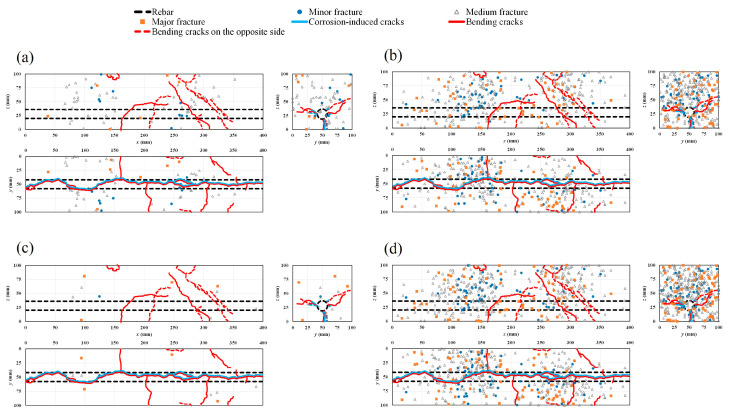
Location of AE sources and crack patterns for 9.3% corrosion level: (**a**) stage I; (**b**) stages II and III; (**c**) stage IV; (**d**) all stages combined.

Figure 12a–d show the progressive ratio of generated AE sources during the bending process with time history for different cases. The results show that in the healthy condition and in all the corroded cases, major damage is quantitatively related to medium fractures. However, the rate of the generated AE sources decreases globally with the increase in the rebar corrosion level, particularly in the vicinity of the failure period. To visualize AE sources inside the specimens, a rectangular prism template model was chosen. Both corrosion-induced cracks and the patterns of bending cracks on different sides of the healthy and corroded specimens are also presented (Figure 13a–e, Figure 14a–e, Figure 15a–e and Figure 16a–d). Those patterns show the interaction between corrosion-induced cracks and bending cracks with the AE sources. In each case, the progress of the generated AE sources during the bending test was presented from stage I up to stage IV of the failure process. To facilitate visualization, the front and back sides were superposed. In the healthy condition and in the case of the low corrosion level of 0.9%, a high rate of AE sources was regularly generated early in stage I, with higher density around the rebar (Figure 13a and Figure 14a). That phenomenon was related to the role of the bond strength between the rebar and concrete, which was not affected by corrosion. A high rate of AE sources was still regularly generated at stages II and III, with the progress of major fracture AE sources toward the failure zones (Figure 13a,b and Figure 14a,b). At stage IV, few AE sources were generated from the rebar location to the top side of the specimens while failure occurred. Major and medium fracture AE sources were dominant during failure (Figure 13d and Figure 14d). However, in the cases of 3.2% and 9.3% corrosion, the trend in the progress of AE sources changed, with far fewer generated at stage I (Figure 15a and Figure 16a). This behavior was related to the weakening of the bond strength between rebar and concrete due to the formation of corrosion around the rebar. However, the density of AE sources decreased with the increase in the corrosion level. In all corroded specimens, the corrosion-induced cracks, which followed a spine line all along the bottom side, were responsible for the initiation of the bending cracks in the tension zone. At the bottom side, new bending cracks were particularly generated following transverse lines. The fluctuation of AE sources at the rebar location during bending tests revealed the condition of the bond strength between rebar and concrete. It decreased in density with the increase in corrosion level. In all cases, the progress of generated AE sources showed a concentration trend around the failure zones.

**Figure 17:**
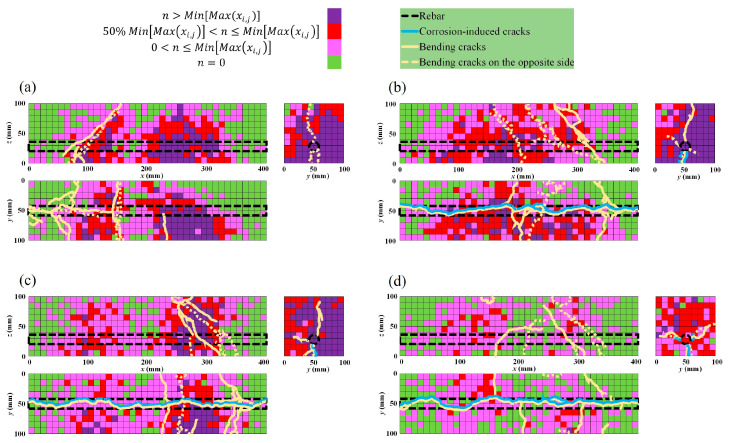
AE sources concentration during bending tests: (**a**) healthy condition; (**b**) 0.9% corrosion level; (**c**) 3.2% corrosion level; (**d**) 9.3% corrosion level.

Furthermore, to evaluate the concentration of AE sources generated during the bending process, a three-dimensional representation was adopted, as presented in Section 3.3.3. The results presented in Table 4 reveal that the maximum concentration of AE sources generated in a cell decreases with the increase in corrosion level. The correlation between the bending cracks and corrosion-induced cracks is illustrated in Figure 17a–d for the four cases. Preexisting rebar damage due to corrosion trends lowers the concentration of AE during the bending failure. In the healthy condition and the low corrosion level of 0.9% (Figure 17a,b), more cells with higher AE source concentration are located in the tension area around the rebar location, where bending cracks were initiated. From the 3.2% to the 9.3% corrosion level, cells with higher AE source concentration migrate progressively toward the compression area at the upper zone of the specimens. That phenomenon describes the impact of preexisting corrosion-induced damages on the bearing capacity of the beams.

## 5. Conclusions

In this study, bending testing up to the failure of small-scale RC beams was carried out under different conditions regarding the progress in rebar corrosion. Acoustic emission measurement was simultaneously performed to evaluate the failure process. The parameters of the recorded AE data were examined for that purpose. Moreover, the effect of preexisting damage caused by rebar corrosion on the bending fracture process could be evaluated as well in concordance to the corrosion level. From the analysis based on the recorded AE parameters during the bending testing, the following main conclusions could be drawn:

- During the bending tests up to failure, the fluctuation of the moving average of the AE maximum amplitudes distinguished four stages of fracture behavior in the healthy condition, in the 0.9% corrosion level, and in the 3.2% corrosion level. These stages were, respectively, related to the nucleation of cracks, the formation of macrocracks from the bottom area of the specimens toward the upper areas, the opening of macrocracks, and the occurrence of failure.
- However, in the case of a severe corrosion level of 9.3%, the fluctuation of the moving average of the AE maximum amplitudes distinguished only three fracture stages because the formation and the opening of macrocracks occurred simultaneously due to the severity of corrosion.
- The mean and the standard deviation of the AE maximum amplitudes allowed the classification of the fractures into minor fractures, medium fractures, and major fractures.
- Based on the three fracture scales, the RA values, and the average frequencies provided a classification of both damage scales and cracks mode (in tensile type and shear-type). Although there is not yet a standard method for fixing the boundary line in the classification of RA values and average frequencies to identify tensile-type and shear-type damages, the method used in this work made possible a comparative analysis of the fracture modes between the healthy condition and the corroded cases.
- Using the digital analysis method proposed in this study, the progress of rebar corrosion could be revealed by the decreasing trend of the concentration of AE sources.

## Figures and Tables

**Table 1:** Mix proportions of concrete.

Water- Cement Ratio (%)	Sand- Aggregate Ratio (%)	kg/m^3^						
		Cement: Ordinary Portland Cement	Sand	Gravel		Water	Water Reducing Agent (C × 0.8%)	Air Entrainment (Diluted 100 Times, C × 0.4%)
				Fine Aggregate (05 mm–15 mm)	Coarse Aggregate (10 mm–20 mm)			
50	46.5	332	808	489	489	166	2.66	0.66
Targeted slump: 12 ± 2.5 cm; Targeted air content: 4.5 ± 1.5%								

**Table 2:** AE sensors’ coordinates on measurement specimens.

Sensor Channel	Coordinates (mm)			Side
	x	y	z	
Ch. 1	65	0	80	Front
Ch. 2	340	0	15	Front
Ch. 3	100	100	25	Back
Ch. 4	290	100	50	Back
Ch. 5	150	30	0	Bottom
Ch. 6	250	75	0	Bottom

**Table 3:** AE maximum amplitude fluctuation interval for minor, medium, and major intervals.

Cases	Parameters		Minor Fracture Amplitudes Range (dB)	Medium Fracture Amplitudes Range (dB)	Major Fracture Amplitudes Range (dB)
	Mean Maximum Amplitude µ (dB)	Standard Deviation *σ*			
Healthy condition	62	9	[40, 52]	[53, 71]	[72, 100]
0.9% corrosion level	60	10	[40, 49]	[50, 70]	[71, 100]
3.2% corrosion level	60	10	[40, 49]	[50, 70]	[71, 100]
9.3% corrosion level	61	10	[40, 50]	[51, 71]	[72, 100]

**Table 4:** Maximum number of AE sources in a cell via the front side of the specimen.

Corrosion Level	$\mathrm{Maximum}\;\mathrm{Number}\;\mathrm{of}\;\mathrm{AE}\;\mathrm{Sources}\;\mathrm{in}\;a\;\mathrm{Cell}\;\mathrm{via}\;\mathrm{the}\;\mathrm{Font}\;\mathrm{Side}\;{Max\left(x_{i,j}\right)}_{1\leq i\leq 40,1\leq j\leq 10}$	$Min\left[Max\left(x_{i,j}\right)\right]$ 1≤i≤40,1≤j≤10
Healthy condition (0%)	34	7
0.9%	17	
3.2%	16	
9.3%	7	

## Data Availability

The authors declare that the raw data supporting the results of this article are available upon request.
